# Supplementary figures and images for: Cryo-EM structures reveal high-resolution mechanism of a DNA polymerase sliding clamp loader
Source: eLife. 2022 Feb 18;11:e74175. doi: 10.7554/eLife.74175 (PMC8893722; doi:10.7554/eLife.74175)

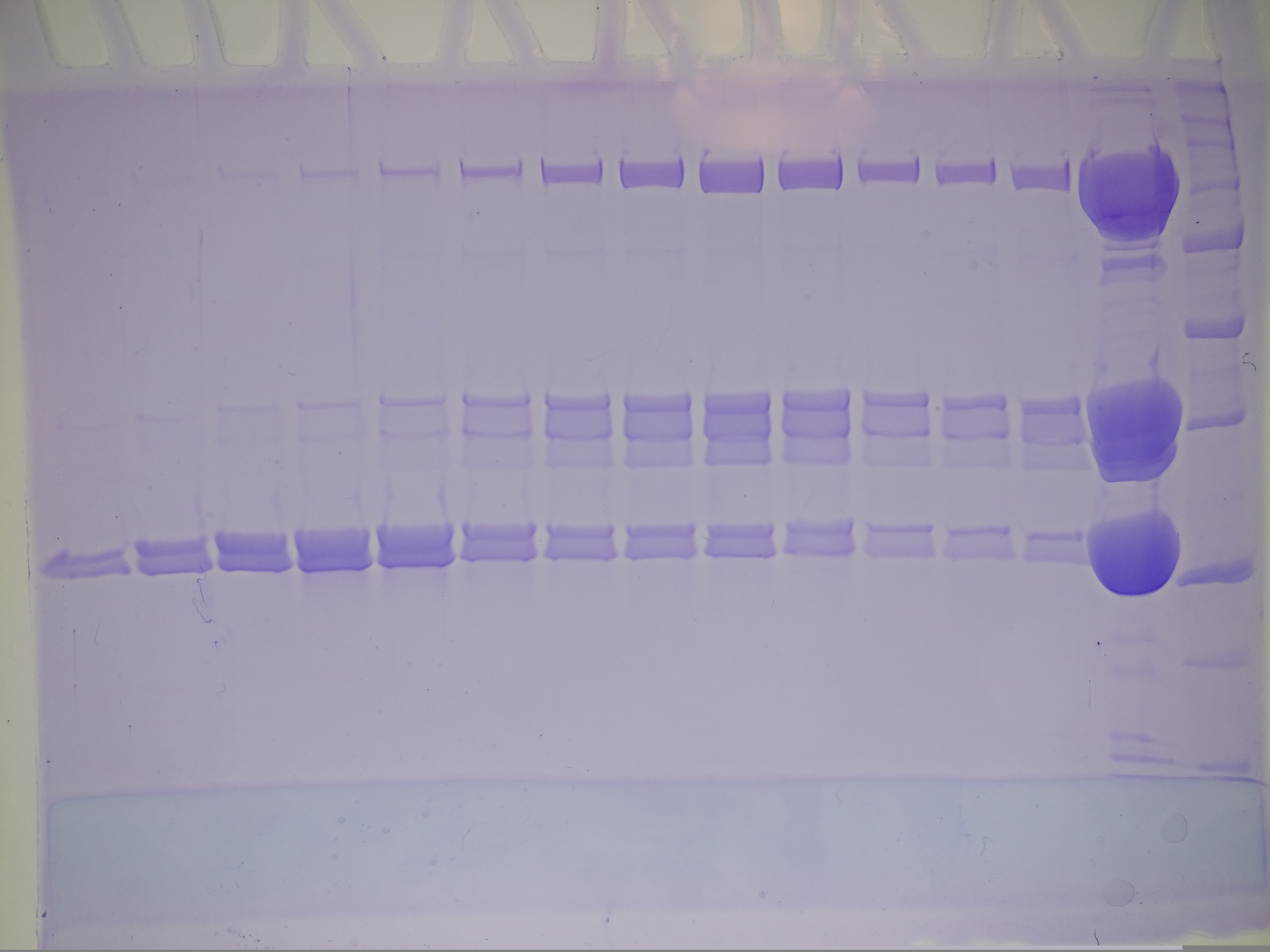

Supplement: Figure 1—figure supplement 1—source data 1. [file elife-74175-fig1-figsupp1-data1.zip › Figure1-FigSupplement1-SourceData1/Figure1-FigureSupplement1A-uncropped.jpg]

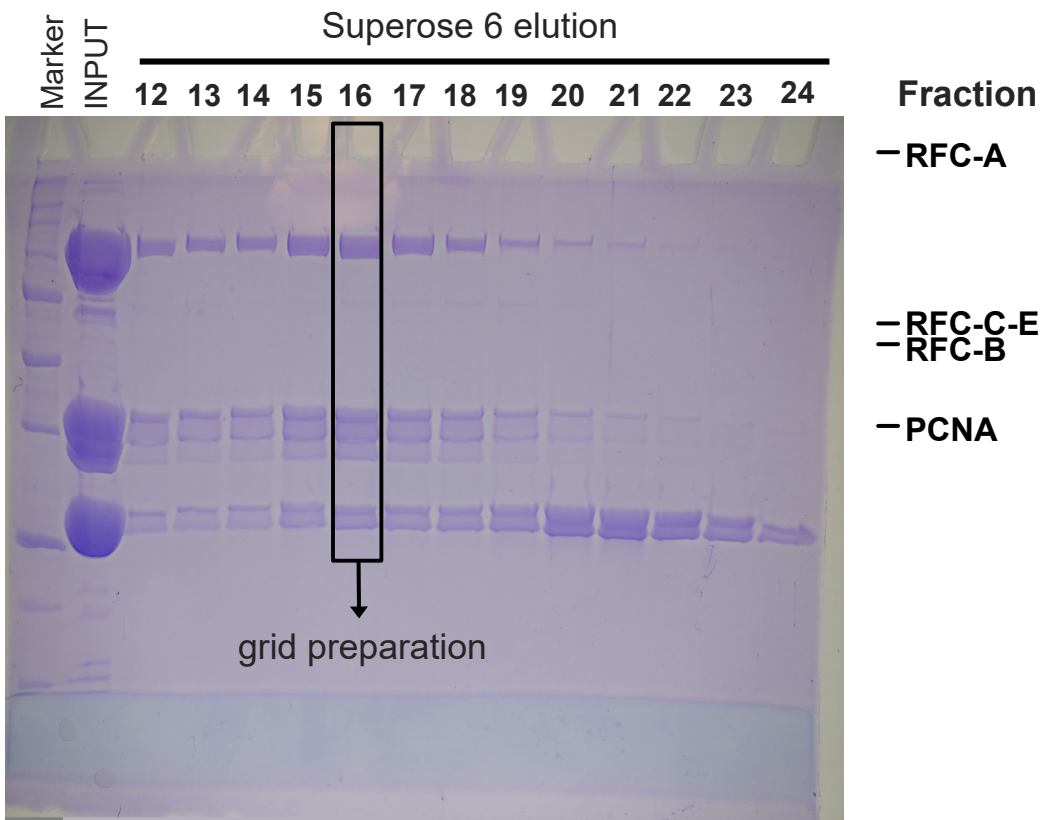

Supplement: Figure 1—figure supplement 1—source data 1. [file elife-74175-fig1-figsupp1-data1.zip › Figure1-FigSupplement1-SourceData1/Figure1-FigureSupplement1A-uncropped.pdf]
